# Molecular Epidemiological Investigation and Viral Isolation of Porcine Rotavirus in Southwest China During 2024–2025

**DOI:** 10.3390/vetsci12121137

**Published:** 2025-11-29

**Authors:** Sirun He, Jiqiang Shi, Huanyuan Hu, Xiaozhen Zhang, Mao Ning, Wensi Wu, Yiping Wen, Yiping Wang, Rui Wu, Qin Zhao, Senyan Du, Sanjie Cao, Xiaobo Huang, Shan Zhao, Yifei Lang, Nanfang Zeng, Qigui Yan

**Affiliations:** 1 College of Veterinary Medicine, Sichuan Agricultural University, Chengdu 611130, China; sirunhe1122@163.com (S.H.); 1192827736@qq.com (H.H.); 2915203827@qq.com (X.Z.); yueliang5189@163.com (Y.W.); yipingwang@sicau.edu.cn (Y.W.); wurui1977@163.com (R.W.); zhao.qin@sicau.edu.cn (Q.Z.); senyandu@sicau.edu.cn (S.D.); csanjie@sicau.edu.cn (S.C.); rsghb110@126.com (X.H.); zhaoshan419@163.com (S.Z.); y_langviro@163.com (Y.L.); 2 Giantstar Farming and Husbandry Co., Ltd., Chengdu 611130, China; 321275019@qq.com (J.S.); ningmaocau@163.com (M.N.); h_u_a_n_g_w_u@foxmail.com (W.W.)

**Keywords:** porcine rotavirus, molecular epidemiology, genotyping, virus isolation, phylogenetic analyses

## Abstract

In 2023, the detection rate of porcine rotavirus (PoRV) surpassed that of porcine epidemic diarrhea virus (PEDV) for the first time, establishing PoRV as the predominant pathogen responsible for viral diarrhea in pigs. To systematically investigate the epidemiology and molecular characteristics of PoRV in Southwest China, a total of 196 diarrheal clinical samples were collected from 29 large-scale pig farms across the region during 2024–2025. RT-qPCR results revealed a high PoRV positivity rate of 57.14% (112/196) with group A porcine rotavirus (PoRVA) being the most prevalent at 46.43%, representing the predominant group. Genotyping and phylogenetic analysis of the VP4 and VP7 genes indicated that the P genotype P[13] was most prevalent (77.78%, 21/27), while the major G genotypes were G4 (39.28%) and G9 (35.71%). The most common G/P combinations were G9P[13] and G4P[13]. Furthermore, a PoRV strain was successfully isolated and identified through whole-genome sequencing, indirect immunofluorescence assay (IFA), and transmission electron microscopy (TEM). The isolate was designated RVA/Pig-wt/SCLS-JW/2024/G1P[7], with a whole-genome constellation of G1-P[7]-I5-R1-C1-M1-A8-N1-T1-E1-H1. The structural proteins VP1-4 and VP6-7, along with nonstructural genes NSP1 and NSP5, shared high sequence identity with porcine strains, whereas the nonstructural genes NSP2–NSP4 clustered more closely with human rotaviruses. These findings indicate a higher prevalence of PoRV in southwestern China compared to other regions; the dominant circulating genotypes have shifted to G9 and G4; the isolated G1P[7] strain is relatively rare in China and might be a genetic recombinant of human and porcine rotaviruses. This study provides valuable data and theoretical support for understanding the current epidemiology of PoRV, and facilitates vaccine development and the formulation of prevention and control strategies.

## Simple Summary

Rotavirus (RV), a major cause of acute diarrhea in young children and animals, frequently leads to co-infections with other diarrheal pathogens, inflicting substantial economic losses on the swine industry. During 2023–2024, 196 clinical diarrheal samples from Southwest China were analyzed, revealing a porcine rotavirus (PoRV) positivity rate of 57.14%. Group A porcine rotavirus (PoRVA) was the most prevalent, with a genotypic shift observed toward G9 and G4, alongside the identification of rotaviruses from diverse origins. The successful isolation of the RVA/Pig-wt/SCLS-JW/2024/G1P[7] strain and subsequent genomic analysis indicated it is a human–porcine recombinant, suggesting cross-species transmission. These findings provide crucial data for understanding PoRV epidemiology and vaccine development, underscoring the need for ongoing rotavirus surveillance.

## 1. Introduction

Rotavirus (RV) is the main pathogen that causes acute diarrhea in children and animals [1]. Since isolation of porcine rotavirus (PoRV) [2] from diarrhea samples of weaned piglets was first reported by Woode et al. in 1975, diarrhea caused by rotavirus infection in piglets has been reported in major swine-producing countries around the world [3,4,5]. In clinical practice, PoRV is frequently co-infected with other diarrheal disease pathogens, such as porcine epidemic diarrhea virus (PEDV) [5,6], porcine delta coronavirus (PDCoV) [5], and transmissible gastroenteritis virus (TGEV) [6], and caused substantial economic losses of the swine industry.

The complete genome of rotavirus is about 18 kilobases (kb) and comprises 11 double-stranded RNA (dsRNA) segments, encoding six viral structural proteins (VPs) VP1-VP4, VP6 and VP7, five or six nonstructural proteins (NSPs) NSP1-NSP6 [7,8,9]. According to the different strains, gene segment 11 of most rotavirus strains encodes two open reading frames (ORFs) for NSP5 and NSP6 [9]. The International Committee on Taxonomy of Viruses (ICTV) categorizes rotaviruses into 10 groups based on VP6 antigen, including group A rotavirus (RVA) to group J rotavirus (RVJ) [10,11]. Among these groups, RVA, RVB, and RVC are most prevalent in humans and animals, with RVA demonstrating the broadest epidemiological distribution. Based on differences in the VP6 antigen, RVA is further classified into genogroups I and II. As of April 2024, a total of 42 G genotypes (G1–G42) have been identified according to the VP7 antigen, and 58 P genotypes (P[1]–P[58]) based on the VP4 antigen.

PoRV has historically received limited attention due to its low mortality rate and previously localized, small-scale outbreaks. However, in recent years, the global prevalence of PoRV has increased markedly, frequently causing severe outbreaks of diarrhea [12]. Based on the statistical method described by Papp et al. [13], the global prevalence of group A porcine rotavirus (PoRVA) groups from 2005 to 2022 across five continents (excluding China) was analyzed. The prevalence rate increased from 23.99% prior to 2004 to 47.02% in the 2005–2022 period. Concurrently, genetic epidemiology has become increasingly complex. While G5 and P[7] were dominant before 2004 [14,15,16], since 2005, multiple G and P genotypes have become highly prevalent. Not only has the proportion of single genotypes significantly decreased, but emerging novel genotypes have also been observed [17,18]. Nevertheless, G3, G4, and G5, along with P[6], P[7], and P[13], remain the most prevalent genotypes globally. In comparison, the overall prevalence of PoRVA in China was relatively low but varied considerably across regions, with Southwest China having the highest prevalence rate (22.11%) and Central China the lowest (3.55%). G3, G4, G5, and G9 are the most common G genotypes in China, P[6], P[7], P[13], and P[23] are the predominant P genotypes, with G9 and P[23] being particularly widespread [19,20,21].

The widespread prevalence of PoRV has expanded the host population, increased the frequency of co-infections, and intensified immune selection pressure, significantly accelerating the virus’s genomic mutation and recombination. Newly emerged recombinant or mutant strains often exhibit enhanced transmission, virulence, and immune evasion capabilities, thereby exacerbating epidemic intensity and complicating disease control efforts. This vicious cycle underpins the current surge in PoRV genotype diversity and the challenge of controlling the virus with monovalent vaccines, posing a serious threat to existing disease prevention strategies and vaccine efficacy. Currently, research on PoRV remains largely focused on pathogen isolation and identification, with a lack of systematic investigation into its genotypic characteristics. Therefore, this study focused on southwestern China, where PoRV prevalence is highest, to investigate the epidemiology and genetic evolutionary relationships of PoRV in large-scale pig farms. A PoRV strain designated RVA/Pig-wt/SCLS-JW/2024/G1P[7] was isolated and subjected to whole-genome sequencing and phylogenetic analysis. This study aims to address current research gaps and provide critical scientific evidence and data support for the development of relevant vaccines and the effective prevention and control of PoRV.

## 2. Materials and Methods

### 2.1. Cells, Antibodies, and Clinical Samples

This study utilized MA104 cells sourced from the College of Veterinary Medicine at Sichuan Agricultural University. The cells were cultured under standard conditions using Dulbecco’s modified Eagle medium (DMEM; Thermo Fisher Scientific Gibco, Waltham, MA, USA) enriched with 10% fetal bovine serum (FBS; ExCell Bio, Suzhou, China). The antibodies used included PoRV-positive porcine serum (College of Veterinary Medicine, Sichuan Agricultural University), fluorescein isothiocyanate (FITC)-conjugated goat anti-porcine IgG (Abcam, Cambridge, UK), and 4′,6-diamidino-2-phenylindole (DAPI; Solarbio, Beijing, China).

Regarding the samples, a collection of 196 specimens (100 intestinal tissues and 96 fecal samples) was gathered between 2024 and 2025 from 29 large-scale farms in Southwest China. These were all preserved at −80 °C for subsequent analysis.

### 2.2. RNA Extraction and Detection of PoRV

The tissue samples were homogenized in sterile phosphate-buffered saline (PBS) at a volume ratio of 1:2. The homogenates underwent three sequential freeze–thaw cycles, followed by centrifugation at 5000 r/min for 15 min. The clarified supernatants were then filtered through a 0.45 μm syringe filter (Biosharp, Hefei, China), aliquoted, and stored at −80 °C for subsequent virus isolation. Viral RNA was extracted using a Viral RNA Kit (Omega Bio-Tek, Inc., Norcross, GA, USA) according to the manufacturer’s instructions.

Referring to the reverse transcription quantitative polymerase chain reaction (RT-qPCR) primers and hydrolysis probes based on the PoRVA, PoRVB, and PoRVC VP6 genes designed by Marthaler et al. [22] (Table 1). RT-qPCR was performed using HiScript II U+ One Step qRT-PCR Probe Kit (Vazyme Biotech Co., Ltd., Nanjing, China) containing UNG enzyme, with a reaction system of 20 μL: 2× One step U Mix 10 μL; One Step U+ Enzyme Mix 1 μL; 0.4 μL of each primer (10 μM); probe 0.2 μL; template RNA 5 μL; H_2_O to 20 μL. Negative controls were set up using nuclease-free water as the template, while positive controls used laboratory-stored RNA from previously sequenced PoRVA, PoRVB, and PoRVC strains. The thermal cycling protocol was as follows: reverse transcription at 50 °C for 15 min, followed by pre-denaturation 95 °C for 5 min, and then 40 cyclics of 95 °C for 10 s, 60 °C for 30 s. The VP6 gene was amplified utilizing a Exlycle-48 Fluorescent Quantitative PCR Instrument (Glinxbio Co., Ltd., Shanghai, China). Confirmation of its amplification curves and melting curves at the end of the reaction.

### 2.3. Amplification and Sequencing of VP4 and VP7 Gene

The VP4 and VP7 genes of PoRVA-positive samples were amplified by one-step reverse transcription polymerase chain reaction (RT-PCR) using specific amplification primers (Table 2). The RT-PCR reaction was performed using HiScript II One Step RT-PCR Kit (Vazyme Biotech Co., Ltd., Nanjing, China) in a total volume of 50 μL, containing 25 μL of 2×One Step Mix, 2.5 μL of One Step Enzyme Mix, 2 μL of each primer (10 μM), 2 μL of template RNA and 16.5 μL of H_2_O. Negative controls were set up using nuclease-free water as the template, while positive controls used laboratory-stored RNA from previously sequenced PoRVA strains. The thermal cycling protocol was as follows: reverse transcription, 30 min at 50 °C; pre-denaturation 3 min at 94 °C; followed by 35 cycles of 94 °C for 30 s, 55 °C for 30 s, and 72 °C for 30 s, as well as a 5 min extension at 72 °C. Then, the RT-PCR products were electrophoresed in 1.5% agarose gels (Yeasen Biotechnology, Shanghai, China) using a Power Pac Universal system (Bio-Rad Laboratories, Hercules, CA, USA), and visualized with a GEL DOC™ XR+ imaging system (Bio-Rad, Hercules, CA, USA). Target fragments were purified using a gel extraction kit (Omega Bio-Tek, Norcross, GA, USA) and the gel extraction fragments were sequenced by Sangon Biotech Co., Ltd. (Shanghai, China).

### 2.4. Virus Isolation and Whole-Genome Amplification

MA104 cells were seeded in 12-well plates for virus isolation. The supernatant from processed positive samples was supplemented with trypsin (Sigma-Aldrich Trading Co., Ltd., St. Louis, MO, USA) to a final concentration of 20 μg/mL. After thorough vortex mixing, the mixture was activated in a 37 °C incubator with 5% CO_2_ for 1.5 h. Following two washes with PBS, the MA104 cells were incubated with the activated supernatant for 1 h under the same conditions. After removing the inoculum, the cells were overlaid with DMEM containing 5 μg/mL trypsin and further incubate at 37 °C in 5% CO_2_. The cytopathic effect (CPE) was monitored dail. Once CPE was observed in over 80% of the cells, the cell culture was harvested and subjected to three freeze–thaw cycles. The lysate was then centrifuged at 12,000 r/min for 15 min, and the resulting supernatant was collected and stored at −80 °C. This supernatant served as the seed stock for subsequent passages. Blind passages were performed until a significant and stable CPE was consistently observed. As described, PoRV genes were amplified by one-step RT-PCR using viral RNA extracted from the cell culture supernatant as the template, with specific primers targeting each PoRV gene (Table 2). The resulting amplification products were purified by agarose gel electrophoresis, and the extracted DNA fragments were sequenced by Sangon Biotech Co., Ltd.

### 2.5. Immunofluorescence Assay

This assay was conducted following the method reported earlier. MA104 cells were seeded in advance on 14 mm glass cover slips placed in 24-well plates (NEST, Wuxi, Jiangsu, China). Subsequently, the cells were inoculated with PoRV. At 12 and 24 h post-infection (hpi), the cells were first rinsed three times with PBST, and then fixed with 4% paraformaldehyde (PFA) for 30 min at room temperature. After fixation, the cells were blocked using a blocking buffer (2% bovine serum albumin (BSA) in 0.05% PBST (0.05% Tween-20 in PBS)) for 30 min at room temperature. Next, PoRV-positive porcine serum, serving as the primary antibody, was added to the cells and incubated for 45 min. Following this incubation period, the cells were washed three times with 0.05% PBST; afterwards, FITC-conjugated goat anti-pig IgG (diluted at 1:200) and 4,6-diamidino-2-phenylindole (DAPI, Solarbio Science & Technology Co., Ltd., Beijing, China) were added, and the mixture was incubated at room temperature for 30 min. Thereafter, the cells were rinsed five times with PBST. The stained cells were observed under a fluorescence microscope (Olympus, Tokyo, Japan).

### 2.6. Electron Microscopy Analysis

To purify the virus, the virus-containing supernatant was first clarified by centrifugation at 12,000 rpm for 30 min at 4 °C to remove cellular debris. A 500 μL sucrose cushion (20%) was then carefully underlaid beneath the supernatant to maintain phase separation. The sample was subjected to ultracentrifugation at 40,000 rpm for 3 h at 4 °C. After discarding the supernatant, the tube was inverted on absorbent paper for 10 min to drain residual liquid. The pelleted viral particles were resuspended in 500 μL of PBS, negatively stained with 2% phosphotungstic acid, and finally observed under a transmission electron microscope (Hitachi, Tokyo, Japan).

### 2.7. Titration and Growth Curve Determination

The growth curve of the 8th generation of the virus was constructed. Initially, the virus was inoculated onto a 24-well plate at a multiplicity of infection (MOI) of 0.01. Cell culture supernatants were collected at 6, 9, 12, 15, 18, 24, and 30 hpi. Subsequently, MA104 cells cultured in 96-well plates. After washing thrice with PBS, 100 μL of the experimental supernatant was mixed with 900 μL of DMEM to obtain serial dilutions ranging from 10^−1^ to 10^−10^. Each dilution was added to a monolayer of MA104 cells in 10 vertical wells of a 96-well plate. The plate was then placed in a cell culture incubator at 37 °C for 72 h, and the stained cells were observed under a fluorescence microscope by IFA. Virus titers were calculated via the Reed–Muench method. Eventually, the growth curve for virus was constructed based on the viral titers at different time points post-infection.

### 2.8. Phylogenetic and Homology Analysis

The best-fit models for PoRV each genomic sequences were selected utilizing Models in MEGA X (Version 10.1.5), and phylogenetic trees were constructed using the maximum likelihood (ML) method. Bootstrap values were estimated for 1000 replicates. The information about the reference strains of PoRV is displayed in Table S1.

## 3. Results

### 3.1. Prevalence of PoRV in Clinical Sample

A total of 196 samples that were collected from 29 large-scale farms of southwestern China were detected using RT-qPCR. The overall positivity rate for PoRV was 57.14% (112/196). Among the 112 positive samples, the rates of mono-infection with PoRVA, PoRVB, and PoRVC in samples tested were 46.43% (52/112), 10.71% (12/112), 33.04% (37/112), respectively. The prevalence rate of co-infections involving two or more groups was 9.82% (11/112). The most common co-infection was PoRVB with PoRVC, with a positivity rate of 7.14% (8/112) (Table 3).

### 3.2. Phylogenetic Analysis of the VP4 and VP7 in PoRV-Positive Samples

From the 52 PoRVA-positive samples, 27 full-length VP4 genes and 28 full-length VP7 genes were successfully amplified. Phylogenetic analysis and homology comparison were performed based on the obtained amino acid sequences.

**Figure 1:**
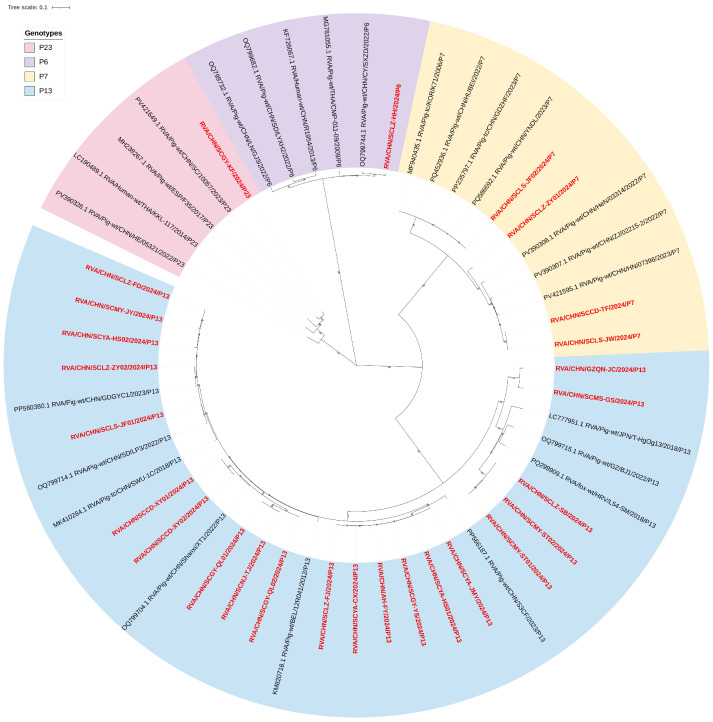
ML trees of the PoRVA samples VP4 gene. Model as GTR + G + I, the sequences collected in this study were marker in red.

The ML tree of VP4 gene illustrated that these sequences could be classified into four P genotypes: P[6], P[7], P[13], and P[23] (Figure 1). Specifically, the ML tree revealed that P[13] as the dominant genotype, accounting for 77.78% (21/27) of the total samples, followed by P[7] at 14.81% (4/27), the least frequent genotypes were P[6] and P[23] accounted for 3.7% (1/27 each).

Within the P[13] genotype samples, four samples (SCLAZ-FD, SCMY-JY, SCYA-HS02, and SCLZ-ZY02) were closely related to RVA/Pig-wt/CHN/GDGYC1, among which SCLZ-ZY02 exhibited the nucleotide homology of 98.63%. Five samples (SCCD-XY01, SCCD-XY02, SCGY-QL01, SCGY-QL02, and SCNJ-TJ) were closely related to RVA/Pig-wt/CHN/XT. Meanwhile, six samples (SCYA-CX, SCLZ-FJ, AH-FY, SCYA-HS01, SCYA-JMY, and SCGY-YS) all formed a close relationship with RVA/Pig-wt/CHN/S3C. SCLS-JF01 was most closely related to RVA/Pig-wt/CHN/LP3, with nucleotide homology of 97%. The remaining five samples exhibited the highest similarity to RVA/Pig-wt/GZ/BJ1. Among the P[7] genotype samples, SCLS-JF02 demonstrated a close relationship with RVA/Pig-wt/CHN/HUBEI, while all remaining samples were most closely related to RVA/Pig-wt/CHN/HN/07396. The single P[6] genotype sample was most closely related to RVA/Pig-wt/CHN/CY/SXZD, with a maximum similarity of 97.65%. Similarly, the single P[23] genotype sample displayed the closest relationship to RVA/Pig-wt/CHN/10057 and the highest similarity of 96.05% (Figure 1 and Figure S1).

**Figure 2:**
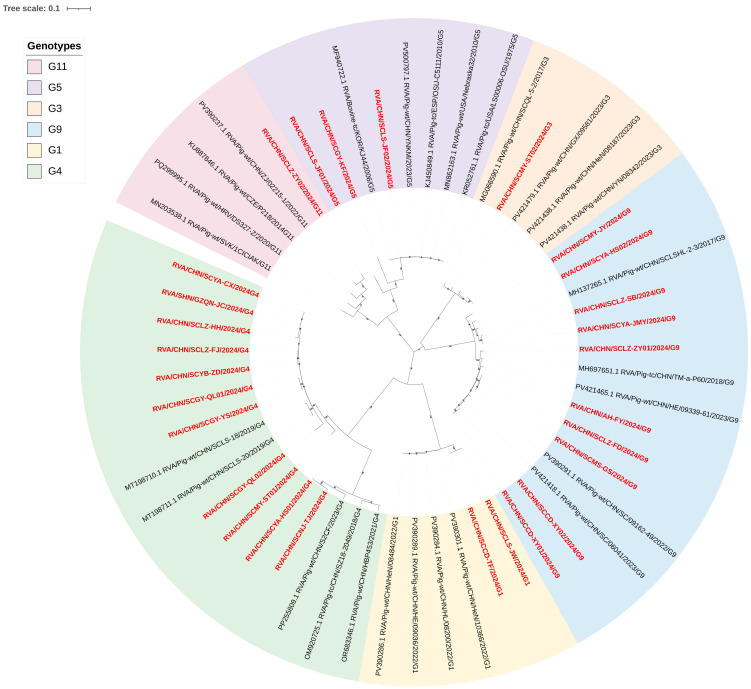
ML trees of the PoRVA samples’ VP7 gene. Model as GTR + G + I, the sequences collected in this study were marker in red.

Based on the ML tree of VP7 gene constructed that these sequences could be classified into six genotypes: G1, G3, G4, G5, G9, and G11 (Figure 2). Genotype distribution analysis revealed G4 as the dominant genotype, accounting for 39.28% (11/28) of the total samples, followed by G9 at 35.71% (10/28), while G5 and G1 accounted for 10.71% (3/28) and 7.14% (2/28), respectively. The least frequent genotypes were G3 and G11, each contributing 3.57% (1/28) to the overall dataset.

Among the G4 genotype samples, except that SCYA-HS01 and SCNJ-TJ shared the closest relationship with RVA/Pig-tc/CHN/S2CF, the remaining G4 samples were more closely related to RVA/Pig-wt/CHN/SCLS-20. Within the G9 genotype samples, four samples (SCMY-JY, SCYA-JMY, SCLZ-SB, and SCYA-HS02) were closely related to RVA/Pig-wt/CHN/SCLSHL-2-3. SCLZ-SZY01 was most closely related to RVA/Pig-tc/CHN/TM-a-P60, with a peak similarity of 97%. The remaining G9 samples were more closely related to RVA/Pig-wt/CHN/SC/06041, with all similarities exceeding 97%. All G5 genotype samples demonstrated a close relationship with the bovine-origin strain RVA/Bovine-tc/KOR/KJ44 from South Korea. G1 genotype samples displayed the closest relationship to RVA/Pig-wt/CHN/HE/1036, with similarities consistently exceeding 96%. For the G3 genotype, the single sample exhibited its closest relationship to RVA/Pig-wt/CHN/SCQL-5-2, reaching a maximum similarity of 96.9%. Finally, the single G11 genotype sample was most closely related to RVA/Pig-wt/CHN/02215-1, with the highest similarity being 94.7% (Figure 2 and Figure S2).

Overall, based on the comprehensive genotyping analysis of the 27 strains successfully sequenced for both VP4 and VP6 genes, G9P[13] and G4P[13] were identified as the dominant genotype combinations, each accounting for 33.33% (9/27). This was followed by G1P[7] at 7.41% (2/27), while the remaining genotypes (G4P[6], G5P[7], G5P[23], G5P[13], G9P[7], G11P[13], G3P[13]) each contributed 3.7% (1/27).

### 3.3. Virus Isolation and Identification

**Figure 3:**
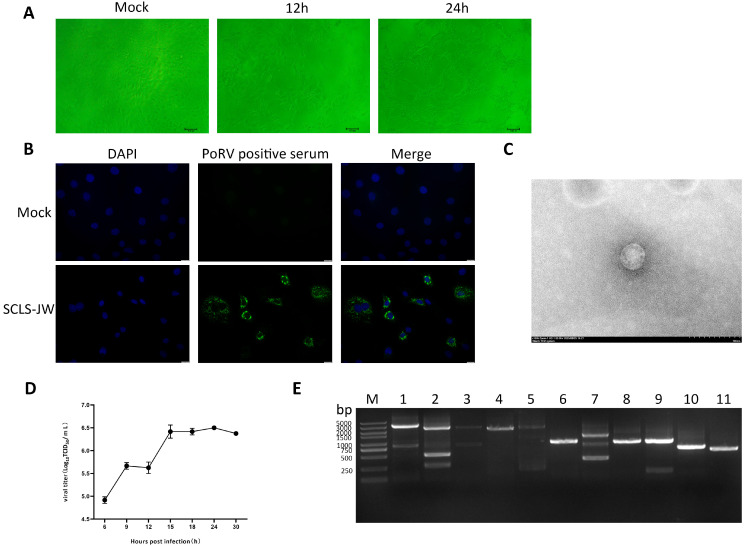
The results of virus isolation and identification (**A**) CPEs induced by PoRV isolate in MA104 cells at 12 and 24 h post-infection (hpi) (100×). (**B**) IFA for PoRV infection in MA104 cells. Viral antigens were detected with a PoRV-positive serum (green), while cell nuclei were counterstained with DAPI (blue) for visualization. (**C**) Electron micrograph of PoRV isolate negatively stained, Scale bar = 100 nm. (**D**) Growth curve of PoRV isolate at in MA104 cells. Data points represent the mean titers from three independent experiments. Error bars depict the standard deviation. (**E**) Amplification results of RT-PCR. M: DNA Marker 5000. 1: PoRV VP1 gene. 2: PoRV VP2 gene. 3: PoRV VP3 gene. 4: PoRV VP4 gene. 5: PoRV VP6 gene. 6: PoRV VP7 gene. 7: PoRV NSP1 gene. 8: PoRV NSP2 gene. 9: PoRV NSP3 gene. 10: PoRV NSP4 gene. 11: PoRV NSP5 gene.

27 PoRVA-positive samples, which both VP4 and VP6 gene sequences were amplified, were inoculated onto MA104 cells. After blind passage to the sixth generation, typical cytopathic effects—including cell detachment, shrinkage, and the formation of neuritic network-like structures—were observed in one positive sample contrast to the uninfected control group (Figure 3A). RT-qPCR confirmation indicated the successful isolation of a PoRV strain, designated PoRV/SCLS-JW/2024.

Furthermore, the isolate was identified using IFA and TEM. IFA performed at 12 h post-inoculation revealed specific green fluorescence in infected cells under a fluorescence microscope, while no signal was detected in the control group (Figure 3B). TEM examination of phosphotungstic acid-negative-stained samples showed round viral particles approximately 70 nm in diameter, covered with short surface projections, consistent with the typical morphology of rotaviruses (Figure 3C). The viral titer of PoRV/SCLS-JW/2024 was determined at different timepoints, reaching a peak of 10^6^ TCID_50_/mL at 24 hpi (Figure 3D). RT-PCR amplification using PoRV segment-specific primers produced amplicons consistent with the expected sizes, as confirmed by agarose gel electrophoresis (Figure 3E).

### 3.4. Phylogenetic Analysis of PoRV/SCLS-JW/2024

The 11 genomic segments of PoRV/SCLS-JW/2024 were subjected to phylogenetic and nucleotide homology analysis alongside reference sequences. The results demonstrated that the isolate corresponds to the genotype constellation G1-P[7]-I5-R1-C1-M1-A8-N1-T1-E1-H1, which is consistent with a typical porcine rotavirus genetic framework. In accordance with the Rotavirus Classification Working Group (RCWG) guidelines, the strain was designated RVA/Pig-wt/SCLS-JW/2024/G1P[7].

**Figure 4:**
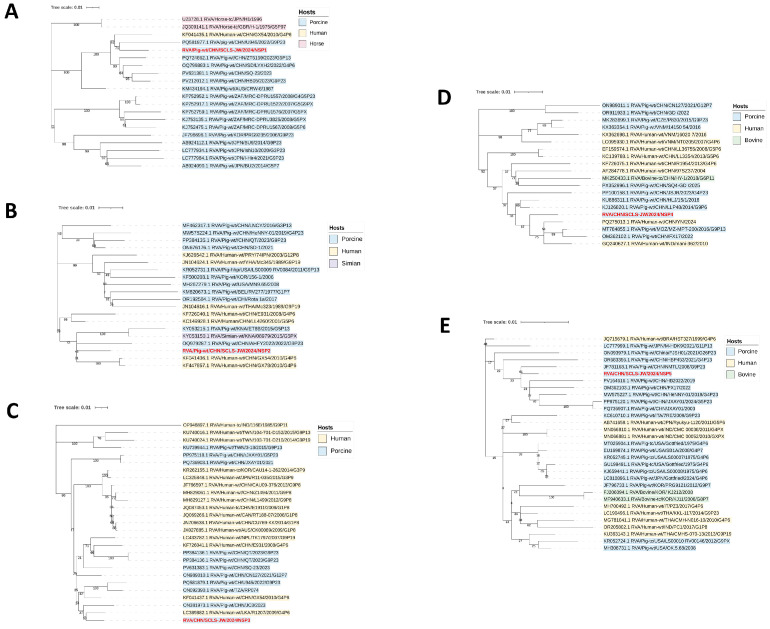
Phylogenetic trees for the NSP1 to NSP5 genes of the RVA/SCLS-JW/2024 isolate are shown in (**A**–**E**). These trees were reconstructed based on the maximum likelihood method implemented in MEGA-X, with branch support assessed by 1000 bootstrap replicates. The corresponding sequences of the RVA/SCLS-JW/2024 isolate are marked in red.

Based on phylogenetic analysis and nucleotide homology comparison, the NSP1 gene was classified as A8 and showed the closest relationship to porcine-origin strain RVA/Pig-wt/CHN/U945 from China (Figure 4A and Figure S3). The NSP2 gene was identified as genotype N1 and was most closely related to the human-origin strain RVA/Human-wt/CHN/GX54 from China, with a nucleotide similarity of 96.85% (Figure 4B and Figure S3). Furthermore, its nucleotide similarity to all human reference sequences exceeded 94%. The NSP3 gene was genotyped as T1 and showed the closest relationship to the human-origin strain RVA/Human-wt/LKA/R1207 from Sri Lanka, with the nucleotide similarity of 97.51% (Figure 4C and Figure S3). The NSP4 gene belonged to genotype E1 and was most closely related to the Chinese human-origin strain RVA/Human-wt/CHN/YN, with a nucleotide similarity of 98.1%, and its nucleotide similarity to all human reference sequences exceeded 95% (Figure 4D and Figure S3). The NSP5 gene was classified as genotype H1 and was most similar to the Chinese porcine-origin strain RVA/Pig-wt/CHN/NMTL, with the nucleotide similarity reaching 98.86% (Figure 4E and Figure S3).

**Figure 5:**
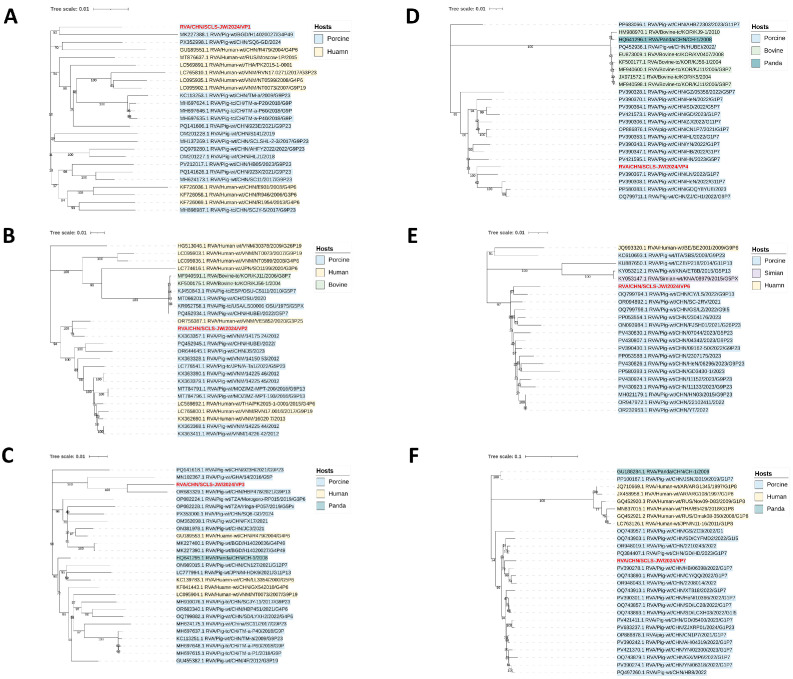
Phylogenetic trees for the VP1 to VP4, VP6, VP7 genes of the RVA/SCLS-JW/2024 isolate are shown in (**A**–**F**). These trees were reconstructed based on the maximum likelihood method implemented in MEGA-X, with branch support assessed by 1000 bootstrap replicates. The corresponding sequences of the RVA/SCLS-JW/2024 isolate are marked in red.

Among the structural genes, VP1 was classified as genotype R1 and showed the closest relationship to the Bangladeshi porcine-origin strain RVA/Pig-wt/BGD/H14020027 (Figure 5A and Figure S4). VP2 was genotyped as C1 and was most closely related to the Vietnamese porcine-origin strain RVA/Pig-wt/VNM/1417524, with a nucleotide similarity of 98.36% (Figure 5B and Figure S4). VP3 was identified as genotype M1 and displayed the highest similarity to the Chinese porcine-origin strain RVA/Pig-wt/CHN/HBP478 (Figure 5C and Figure S4). VP4 gene was genotyped as P[7] and was most closely related to the Chinese porcine-origin strain RVA/Pig-wt/CHN/LN (Figure 5D and Figure S4). VP6 was classified as genotype I5 and shared the closest evolutionary relationship with the Chinese porcine-origin strain RVA/Pig-wt/CHN/CY/LS (Figure 5E and Figure S4). VP7 was identified as genotype G1 and demonstrated the closest phylogenetic affinity to the Chinese porcine-origin strain RVA/Pig-wt/CHN/HB/06398 (Figure 5F and Figure S4).

## 4. Discussion

Viral infection-induced porcine diarrheal diseases inflict substantial economic losses on the swine industry. Previous studies have indicated that PoRV has superseded PEDV as the primary pathogen responsible for viral diarrhea in swine [12]. PoRV exhibits diverse and complex genotype combinations with generally weak cross-protection between different genotypes, leading to highly variable epidemic patterns. Although PoRV prevalent genotypes have undergone considerable alterations, current epidemiological analyses still largely rely on historical data, creating a critical knowledge gap in understanding the current epidemic landscape and formulating effective control strategies, thereby limits timely and scientific guidance for optimizing vaccine strategies and controlling emerging strains. This study was designed to address this gap.

Between 2024 and 2025, we collected 196 samples from 29 large-scale farms in southwestern China. RT-qPCR was performed using group-specific VP6 gene-specific primers to assess the current prevalence of PoRV. The results showed a PoRV positivity rate of 57.14% (112/196) among the samples. Compared with other regions in China, such as Guangdong (36.44%, 289/793), Guangxi (37.13%, 186/501), Jiangxi (38.98%, 145/372), and Fujian (30.49%, 25/82) [23,24], the infection situation in the southwest was more severe and more prevalent, underscoring its role as a major pathogen of porcine diarrhea in this region. Among the 112 positive samples, PoRVA accounted for 46.43% (52/112), PoRVB for 10.71% (12/112), and PoRVC for 33.04% (37/112), indicating that group A is the dominant genotype of PoRV in southwestern China, consistent with reports from other parts of the country during the same period.

To address the crucial need for current genotypic data, this study conducted specific amplification and sequencing of VP4 and VP7 genes from the predominantly circulating group A positive samples. According to VP4 gene phylogenetic analysis, the 27 strains were classified into four P genotypes: P[6], P[7], P[13], and P[23]. The P[13] genotype was the most prevalent at 77.78% (21/27). Phylogenetic analysis based on the VP7 gene revealed six G genotypes: G1, G3, G4, G5, G9, and G11. G4 was the most common (39.28%, 11/28), followed by G9 (35.71%, 10/28). In terms of G/P combinations, G9P[13] and G4P[13] were the predominant genotypes, each accounting for 33.33% (9/27). This finding clearly indicates a shift in the predominant G genotypes, from the previously common G5 type to the currently dominant G9 and G4 types. This genotypic shift has direct implications for vaccine efficacy. The currently available commercial triple live vaccine contains only the G5 genotype [24], and the attenuated live vaccine strain used is also of the G5 type [25], creating a substantial mismatch with the prevailing G9 and G4 genotypes. Thus, current vaccination strategies might provide suboptimal protection, suggesting one potential explanation for the persistent PoRV circulation in Southwestern China. Our findings provide compelling evidence for updating vaccine compositions to include G9 and G4 genotypes. Importantly, all detected G5 strains were most closely related to bovine rotaviruses, suggesting the presence of rotaviruses from diverse origins within Chinese swine herds and necessitating strengthened continuous monitoring of rotaviruses. This study has certain limitations. Due to the limited reference sequences for PoRVB and PoRVC, obtaining their full-length genomic sequences is challenging. Thus, this study focused only on the systematic analysis of 52 PoRVA-positive samples and could not fully reveal the distribution of all prevalent PoRV genotypes in the southwest. Nevertheless, the detailed genotypic data obtained for the predominant PoRVA strains provide substantial value for ongoing PoRV surveillance and vaccine development efforts.

Beyond updating the epidemiological profile, this study also contributes to understanding PoRV diversity and evolution. In this study, a PoRV strain was successfully isolated, the strain was designated RVA/Pig-wt/SCLS-JW/2024/G1P[7]. Whole-genome phylogenetic analysis showed that the isolate has a genotype of G1-P[7]-I5-R1-C1-M1-A8-N1-T1-E1-H1. Previous studies have reported successful isolation of PoRV strains from multiple provinces in China, with G9 genotype being the most frequently isolated, followed by G5, G3, and G4. The predominant P genotypes were P[7] and P[23], with P[13] being less common. Among the common G/P combinations, G9P[23] was the most prevalent, followed by G9P[7], G5P[7], and G3P[13]. This study isolated a G1P[7] PoRV strain in Sichuan Province, which is relatively rare in China, these finding provide new data on the diversity of epidemic PoRV strains in southwestern China. Moreover, the RVA/Pig-wt/SCLS-JW/2024/G1P[7] genome was identified as a recombinant of human (NSP2, NSP3, NSP4) and porcine (NSP1, NSP5, VP1–VP4, VP6, VP7) rotavirus gene segments. The isolation and analysis of this uncommon strain not only enriches the database of circulating PoRV strains in China but also provides important evidence for cross-species transmission events, highlighting the necessity for continuous monitoring of rotavirus evolution.

## 5. Conclusions

This study reveals that the PoRV epidemic in southwestern China is characterized by exceptionally high prevalence and dominance of G9 and G4 strains. The identified mismatch between currently dominant strains and vaccine components, combined with the discovery of rare interspecies recombinant strains, provides critical scientific evidence necessary to inform the development of updated vaccines and optimized control strategies, thereby contributing to the effective prevention and control of PoRV in southwestern China.

## Figures and Tables

**Table 1:** PoRVA, PoRVB, and PoRVC primer and hydrolysis probes.

Primer	Sequence
PoRVA-VP6	F:5′-GCTAGGGAYAAAATTGTTGAAGGTA-3′ R:5′-ATTGGCAAATTTCCTATTCCTCC-3′ Probe: 5′-FAM-ATGAATGGAAATGAYTTTCAAAC-MGB-3′
PoRVB-VP6	F: 5′-GGTTTAAATAGCCCAACCGGTG-3′ R: 5′-TGCAATTTRATGCATGCGTT-3′ Probe: 5′-FAM-AGCATGGATCTGATYGAAACRGT-MGB-3′
PoRVC-VP6	F: 5′-ATGTAGCATGATTCACGAATGGG-3′ R: 5′-ACATTTCATCCTCCTGGGGATC-3′ Probe: 5′-FAM-GCGTAGGGGCAAATGCGCATGA-MGB-3′

**Table 2:** Specific amplification primers.

Primer	Sequence	Length (bp)
PoRV-VP1	F: 5′-GGCTATTAAAGCTGTACAATGG-3′ R: 5′-GGTCACATCTAAGCGCTC-3′	3302
PoRV-VP2	F: 5′-GGCTATTAAAGGCTCAATGG-3′ R: 5′-GGTCATATCTCCACAGTGG-3′	2708
PoRV-VP3	F: 5′-GGCTATTAAAGCAGTACTAGTAG-3′ R: 5′-GGTCACATCGTGACTAGT-3′	2591
PoRV-VP4	F: 5′-GGCTATAAAATGGCTTCGCTCA-3′ R: 5′-GGTCACAACCTCTAGACACTACT-3′	2081
PoRV-VP6	F: 5′-GGCTTTTAAACGAAGTCTTCG-3′ R: 5′-GGTCACATCCTCTCACTA-3′	1356
PoRV-VP7	F: 5′-GGCTTTAAAAGAGAGAATTTCC-3′ R: 5′-GGTCACATCATACAATTCTAATCT-3′	1061
PoRV-NSP1	F: 5′-GGCTTTTTTTATGAAAGGTCTT-3′ R: 5′-GGTCACATTTTATGCTGC-3′	1565
PoRV-NSP2	F: 5′-GGCTTTTAAAGCGTCTCAG-3′ R: 5′-GGTCACATAAGCGCTTTC-3′	1059
PoRV-NSP3	F: 5′-GGCTTTTAATGCTTTTCAGTGG-3′ R: 5′-GGTCACATAACGCCCCTAT-3′	1074
PoRV-NSP4	F: 5′-GGCTTTTAAAAGTTCTGTTCC-3′ R: 5′-GGTCACACTAAGACCATTC-3′	750
PoRV-NSP5	F: 5′-GGCTTTTAAAGCGCTACA-3′ R: 5′-GGTCACAAAACGGGAGTG-3′	667

**Table 3:** The positive rate of distinct PoRV groups.

Groups	A	B	C	Co-Infections			
				A + B	A + C	B + C	A + B + C
Positive Samples (Copies)	52	12	37	1	1	8	1
Positive Rate (%)	46.43	10.71	33.04	0.89	0.89	7.14	0.89

## Data Availability

In this study, the PoRV whole genome sequence are deposited in the NCBI repository, BioProject ID: PRJNA1313801.

## Supplementary Materials

The following supporting information can be downloaded at: https://www.mdpi.com/article/10.3390/vetsci12121137/s1, Figure S1: Nucleotide homology analysis of the VP4 gene of PoRVA samples; Figure S2: Nucleotide homology analysis of the VP7 gene of PoRVA samples; Figure S3: Nucleotide homology analysis of the NSP1–NSP5 gene of PoRV/SCLS-JW/2024; Figure S4: Nucleotide homology analysis of the VP1–VP4, VP6, VP7 gene of PoRV/SCLS-JW/2024; Table S1: The information about the PoRV reference sequences.
