# Supplementary figures and images for: Molecular Epidemiological Investigation and Viral Isolation of Porcine Rotavirus in Southwest China During 2024–2025
Source: Vet Sci. 2025 Nov 29;12(12):1137. doi: 10.3390/vetsci12121137 (PMC12737377; doi:10.3390/vetsci12121137)

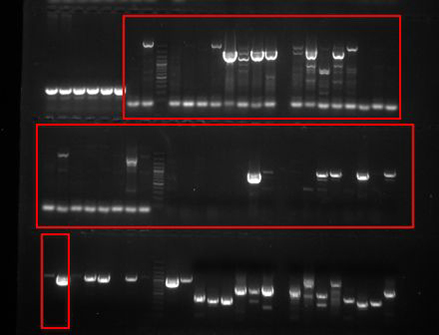

Supplement: Supplementary file 1 [file vetsci-12-01137-s001.zip › PCR/VP4-Original PCR.jpg]

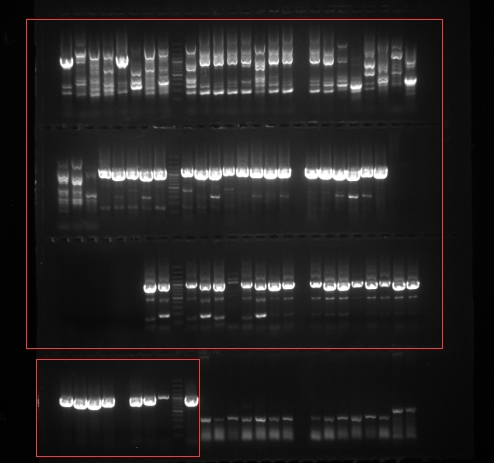

Supplement: Supplementary file 1 [file vetsci-12-01137-s001.zip › PCR/VP4-Original PCR.png]

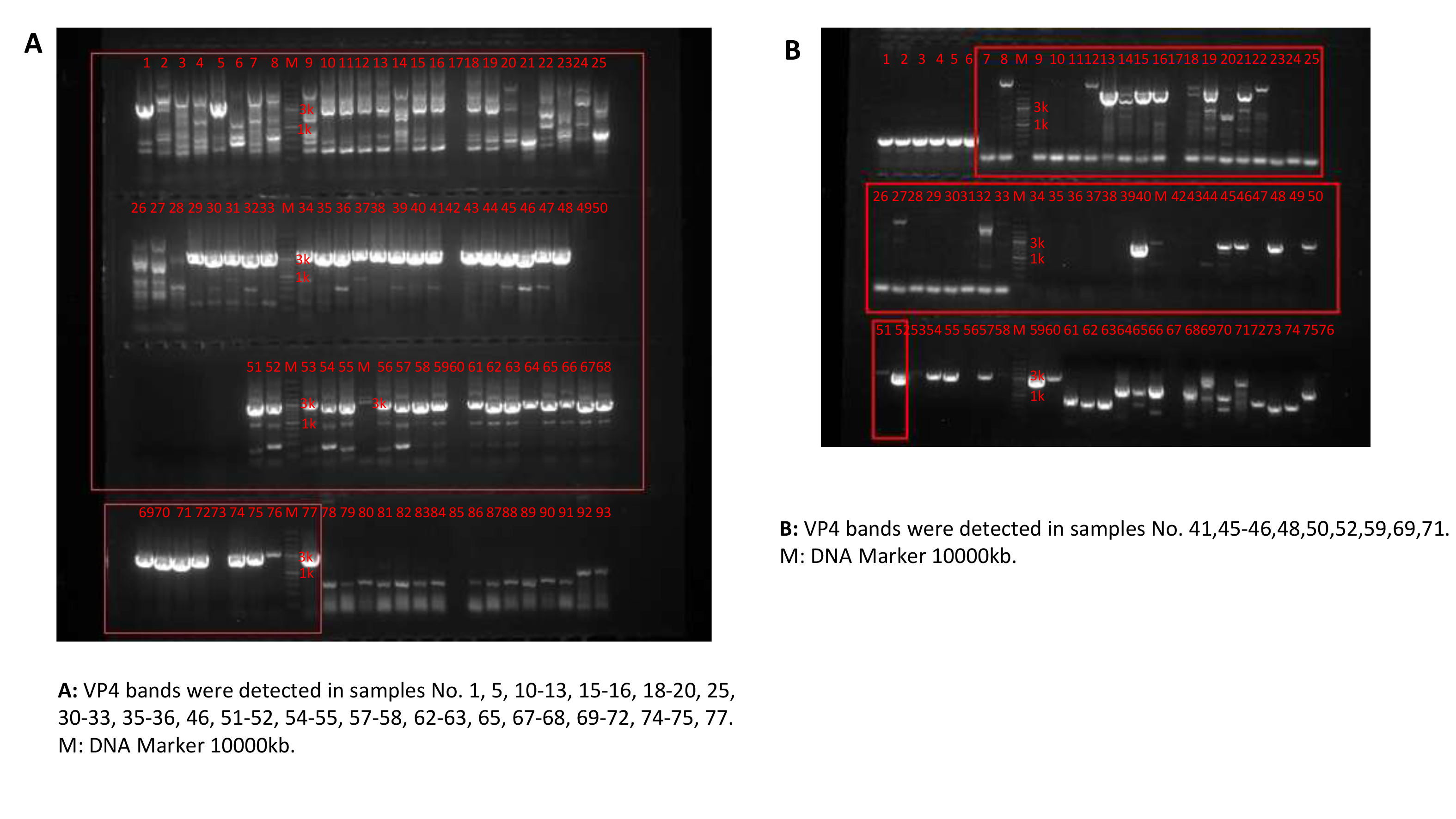

Supplement: Supplementary file 1 [file vetsci-12-01137-s001.zip › PCR/VP4-PCR.jpg]

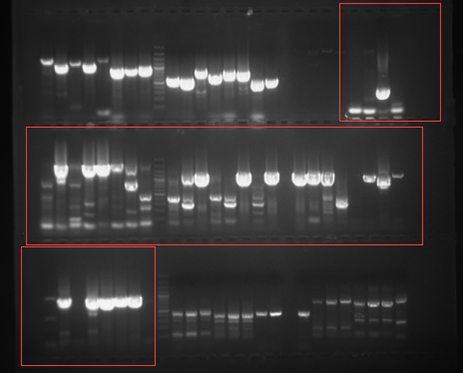

Supplement: Supplementary file 1 [file vetsci-12-01137-s001.zip › PCR/VP7-Original PCR.jpg]

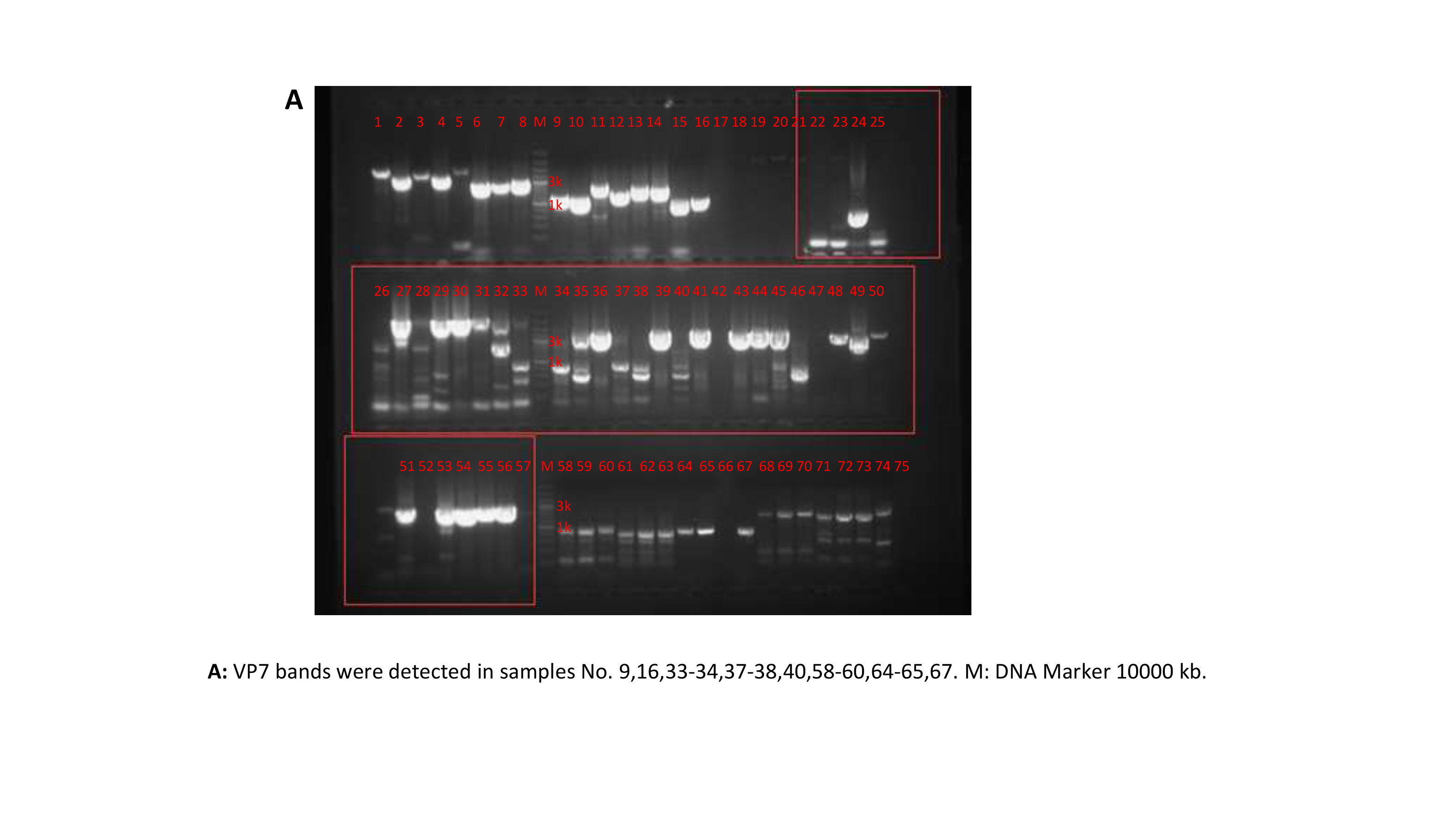

Supplement: Supplementary file 1 [file vetsci-12-01137-s001.zip › PCR/VP7-PCR.jpg]

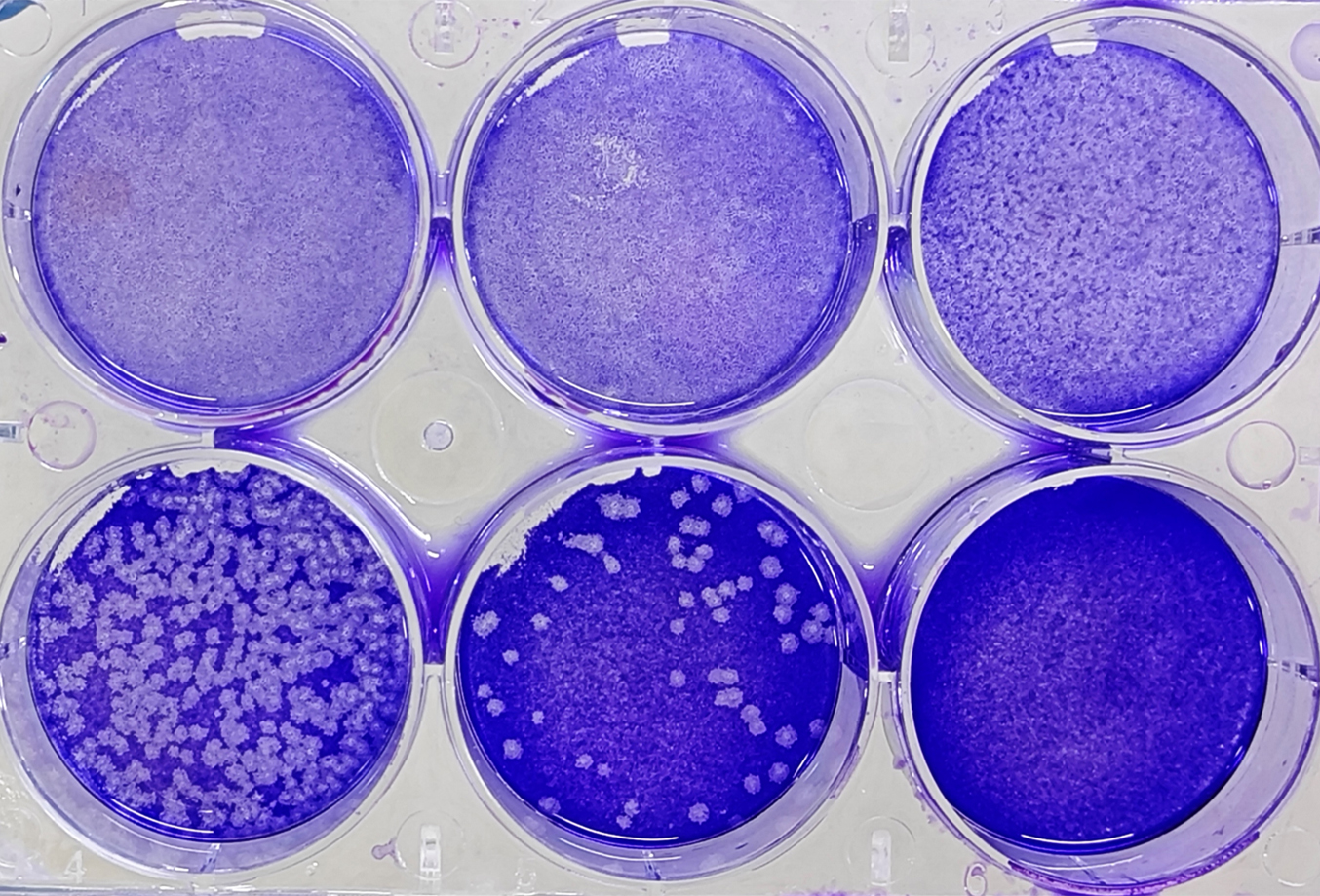

Supplement: Supplementary file 1 [file vetsci-12-01137-s001.zip › plaque purification .jpg]
